# An Ingenane-Type Diterpene from *Euphorbia kansui* Promoted Cell Apoptosis and Macrophage Polarization via the Regulation of PKC Signaling Pathways

**DOI:** 10.3390/ijms251810123

**Published:** 2024-09-20

**Authors:** Xiaoyi Feng, Lizhong Wang, Li Pu, Jianchun Li, Hongmei Li, Dan Liu, Rongtao Li

**Affiliations:** 1 Center for Pharmaceutical Sciences and Engineering, Faculty of Life Science and Technology, Kunming University of Science and Technology, Kunming 650500, China; 2 School of Basic Medical Sciences, Yunnan University of Chinese Medicine, Kunming 650500, China

**Keywords:** *Euphorbia kansui*, ingenane-type diterpenes, anti-proliferation, macrophage polarization, PKC-δ/ERK signaling pathways

## Abstract

*Euphorbia kansui*, a toxic Chinese medicine used for more than 2000 years, has the effect of “purging water to promote drinking” and “reducing swelling and dispersing modules”. Diterpenes and triterpenes are the main bioactive components of *E. kansui*. Among them, ingenane-type diterpenes have multiple biological activities as a protein kinase C δ (PKC-δ) activator, which have previously been shown to promote anti-proliferative and pro-apoptotic effects in several human cancer cell lines. However, the activation of PKC subsequently promoted the survival of macrophages. Recently, we found that 13-hydroxyingenol-3-(2,3-dimethylbutanoate)-13-dodecanoate (compound A) from *E. kansui* showed dual bioactivity, including the inhibition of tumor-cell-line proliferation and regulation of macrophage polarization. This study identifies the possible mechanism of compound A in regulating the polarization state of macrophages, by regulating PKC-δ-extracellular signal regulated kinases (ERK) signaling pathways to exert anti-tumor immunity effects in vitro, which might provide a new treatment method from the perspective of immune cell regulation.

## 1. Introduction

*Euphorbia kansui* has been used for more than 2000 years, mainly for the treatment of esophageal cancer, lung cancer, cancerous melanoma, asthma, and ascites [1,2]. The main bioactive components of *E. kansui* are diterpenes and triterpenes [3,4], which are also its main toxic components. The effectiveness of toxic components and activities of drug-oriented compounds is of great significance to explore, to provide a theoretical basis for the clinical application [5]. Among them, ingenane-type diterpenes have multiple biological activities, such as multidrug resistance reversal activity [4], anti-tumor [6], anti-epilepsy, anti-viral [7], and anti-inflammatory activity [8], lowering cholesterol, protecting the liver and kidney, and scavenging free radicals [9].

**Figure 1:**
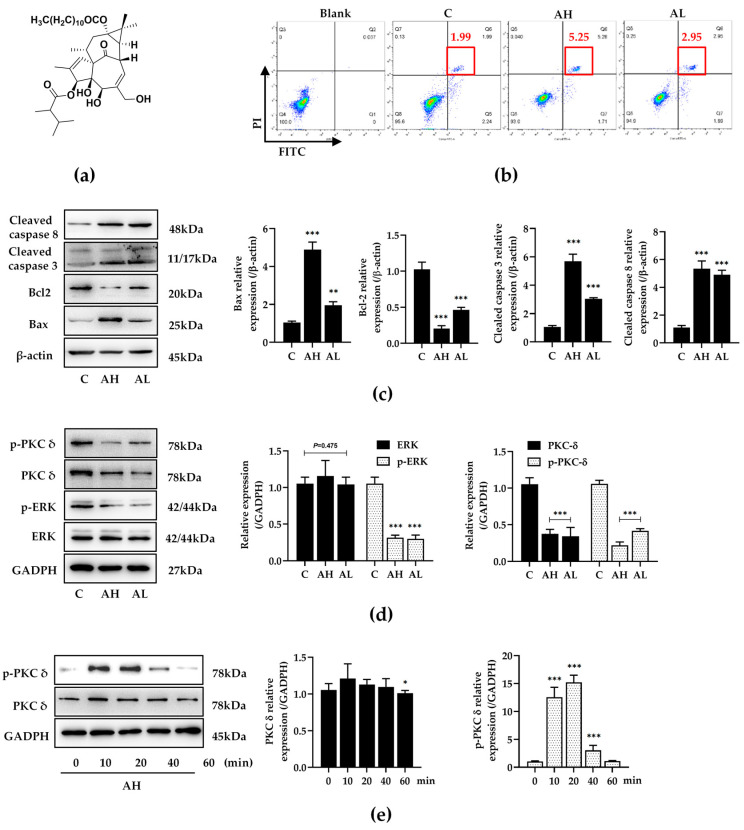
Compound A promoted HepG2 apoptosis and regulated the PKC–ERK signaling pathway. (**a**) Structure of compound A. (**b**) The flow cytometry image of cell apoptosis was double stained with Annexin V and PI. Early apoptotic cells were stained with Annexin V, and late apoptotic cells were stained with PI. The red box represents double staining with Annexin V and PI, and the red text represents the proportion of double stained cells. C: 1‰ dimethyl sulfoxide (DMSO) as a control group; AH: 25 μmol·L^−1^ compound A; AL: 12.5 μmol·L^−1^ compound A. (**c**) The apoptosis-related protein expression was detected by western blotting. ** *p* < 0.01, *** *p* < 0.001 versus the C group. (**d**) The protein expression and phosphorylation of PKC δ (p-PKC δ) and ERK (p-ERK) were detected by western blotting. Data are expressed as the mean ± standard deviation, *** *p* < 0.001 versus the C group. (**e**) The protein expression and phosphorylation of PKC δ and ERK were detected by western blotting after treating with compound A for 0, 10, 20, 40, 60 min. Data are expressed as the mean ± standard deviation, * *p* < 0.05, *** *p* < 0.001 versus the 0 min group.

Diterpenoids have shown significant anti-proliferative and proapoptotic effects in several human cancer cell lines [3,10], such as the breast cancer cell line MCF-7, the liver cancer cell line HepG2, the cervical cancer cell line Hela, the lung cancer cell line A549, etc. The antitumor mechanism is thought to be related to the activation of protein kinase C (PKC), which then triggers apoptosis [11,12]. The PKC signaling pathway is involved in tyrosine kinase-dependent cellular signal transduction [13]. As downstream-signaling molecules, mitogen-activated protein kinase (MAPK) play an important role in a series of defined proliferation, differentiation, and apoptosis events [14]. However, contrary to its proapoptotic effect, PKC played a different role in macrophage behavior in this study [15,16]. The activation of PKC subsequently promoted inducible nitric oxide synthase (iNOS) expression and nitric oxide (NO) release in macrophages [17]. Recently, we found that 13-hydroxyingenol-3-(2,3-dimethylbutanoate)-13-dodecanoate (compound A, the structure is shown in Figure 1a), and ingenane-type diterpenes from *E. kansui* showed anti-cancer and anti-virus bioactivity [18,19]. In addition, our study showed dual bioactivity of compound A, including the inhibition of tumor cell proliferation and regulation of macrophage polarization. Compound A regulated the balance of M1 and M2 phenotypes in macrophages and exhibited probable immunity activation and anti-inflammatory activity in vitro.

The present research is focused on the in-depth understanding of compound A bioactivity involved in the regulation of macrophage polarization and their underlying mechanisms in vitro. It would help to explain the opposite pharmacological effect of ingenane-type diterpenoids on macrophage polarization.

## 2. Results

### 2.1. Bioactivity of Compound A from E. kansui

Compound A was obtained from *E. kansui*, which was identified by Dr. Jianchun Li, Kunming University of Science and Technology. The 1H NMR and 13C NMR spectral data of compound A was supported in supplementary, and data were reported in the literature [4]. Compound A showed inhibitory activity against tumor cells. The half-maximal inhibitory concentrations (IC_50_) of compound A were 21.97 ± 5.01 μM, 27.12 ± 3.34 μM, and 20.97 ± 4.53 μM in A549, MCF-7, and HepG2 cells, respectively (Table 1). The IC_50_ of compound A was 20.34 ± 3.62 μM in the macrophage cell line RAW264.7 (Table 2). Compound A showed a significant inhibitory activity on NO production in lipopolysaccharide (LPS)-stimulated RAW264.7 cells, which was 34 -times higher than that of the positive control drug (L-NMMA). According to the results, the effects of compound A on HepG2 and RAW264.7 cells are discussed for the following experiments.

### 2.2. Inhibition of the Proliferation and Migration of HepG2 Cells via Inhibition of the PKC–ERK Signaling Pathways

The apoptosis events of HepG2 cells were detected by Annexin V/PI staining and Western blotting. The results showed that compound A (12.5 μmol·L^−1^ or 25 μmol·L^−1^) significantly induced apoptosis in HepG2 cells (Figure 1b), and increased the expression of Bax, cleaved caspase 3, and cleaved caspase 8 (Figure 1c). These findings indicate that compound A could accurately induce apoptosis. The expression and phosphorylation of PKC and ERK were detected. The results showed that compound A not only downregulated the phosphorylation of PKC δ (p-PKC δ) and ERK (p-ERK), but also decreased the expression of PKC δ (Figure 1d). As a PKC activator, compound A quickly activated PKC δ, and the phosphorylation of PKC δ was at its highest level in 10–20 min. Compound A then downregulated the expression and phosphorylation of PKC δ over time (Figure 1e), finally inducing the depletion of PKC δ.

### 2.3. Compound A Inhibited M1 Polarization of Macrophages

#### 2.3.1. Compound A Inhibited PKC δ, ERK, and NF-κB Activation of M1 Macrophages

**Figure 2:**
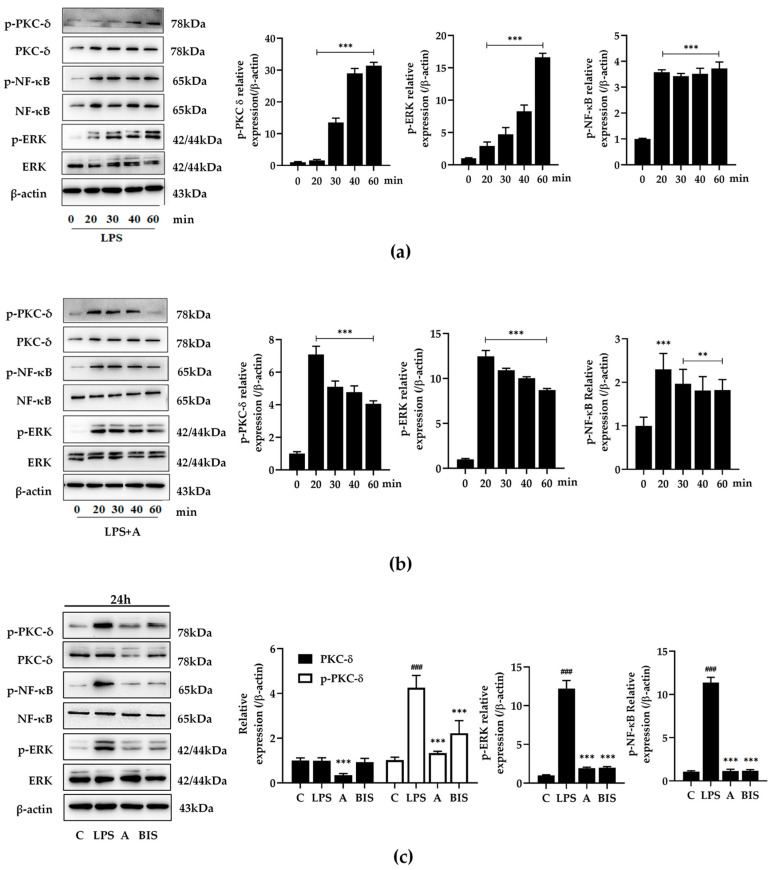
Compound A regulated PKC δ signaling pathways in LPS-stimulated RAW264.7 cells. The expression and phosphorylation of PKC δ, ERK, and NF-κB in RAW264.7 cells was detected by Western blotting after LPS (1 μg·mL^−1^) treatment (**a**) or compound A (10 μmol·L^−1^) and LPS (1 μg·mL^−1^) treatment (**b**) for 0, 20, 30, 40, 60 min. Data are expressed as mean ± standard deviations. Statistical significance was represented as follows: *** *p* < 0.001 versus 0 min group in (**a**). ** *p* < 0.01, *** *p* < 0.001 versus 0 min group in (**b**). (**c**) RAW264.7 cells were exposed to LPS (1 μg·mL^−1^) or compound A or BIS for 24 h, then protein expression was detected. C: 1‰ dimethyl sulfoxide (DMSO) as a control group; LPS: 1 μg·mL^−1^; A: 1 μg·mL^−1^ LPS, and 5 μmol·L^−1^ compound A; BIS: 1 μg·mL^−1^ LPS and 150 nmol·L^−1^ bisindolylmaleimide (BIS). Data are expressed as mean ± standard deviations. Statistical significance was represented as follows: ^###^ *p* < 0.01 versus C group; *** *p* < 0.001 versus LPS group.

Contrary to the effect of PKC δ as a proapoptotic mediator in HepG2 cells, in some circumstances, PKC plays a key role in the expression of internal related genes and the phosphorylation of proteins. In M1 macrophages, PKC δ, ERK, and NF-κB were LPS-stimulated activated with time (Figure 2a), compound A obviously promoted the phosphorylation of PKC δ, ERK, and NF-κB, which reached its highest point at 20–30 min, and then the phosphorylation decreased gradually with time (Figure 2b). Based on this, the phosphorylation of PKC δ, ERK, and NF-κB were investigated for 24 h after being treating with compound A (10 μmol·L^−1^). Compared to the LPS group, compound A and PKC inhibitor (BIS) significantly reduced the phosphorylation of PKC δ, ERK, and NF-κB. Notably, compound A decreased the level of PKC δ protein, but BIS did not. These results indicate that compound A could induce the activation of PKC δ, then be gradually exhausted, which might be its key role in the regulation of downstream signaling pathways.

#### 2.3.2. Compound A Showed Significant Anti-Inflammatory Activity on LPS-Induced Macrophages

**Figure 3:**
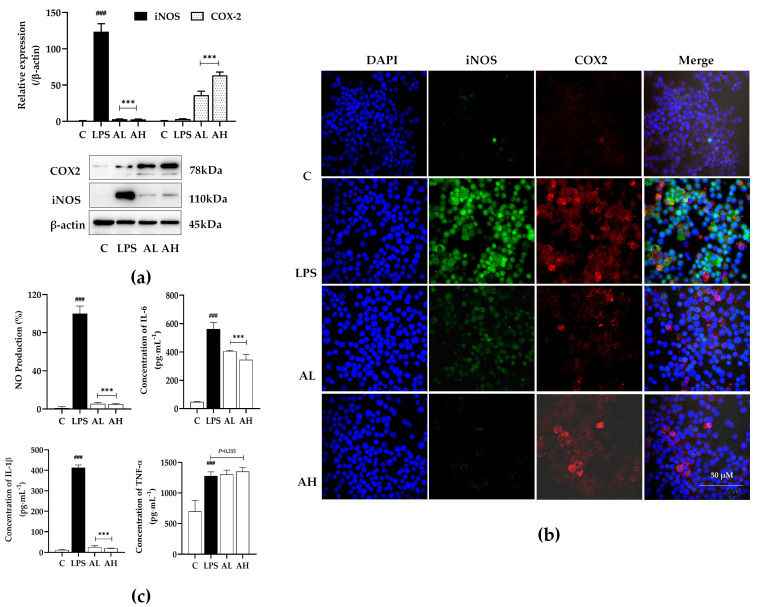
The effects of compound A on the expression of inflammatory mediator by LPS-stimulated RAW264.7 cells. Cells were treated with different concentrations of compound A (10 and 5 μmol∙L^−1^) for 24 h following treatment of LPS (1 μg·mL^−1^), the proteins were detected by Western Blot (**a**) and the cytokines were detected by ELISA (**c**). C: 1‰ dimethyl sulfoxide (DMSO) as control group; LPS: 1 μg·mL^−1^; AL: 5 μmol·L^−1^ compound A; AH: 10 μmol·L^−1^ compound A. (**b**) Representative images of triple immunofluorescence staining of COX-2 (red), iNOS (green) and DAPI (blue) of LPS-stimulated RAW264.7 cells for 24 h. Data are expressed as mean ± standard deviation. Statistical significance was represented as follows: ^###^ *p* < 0.001 versus C group; *** *p* < 0.001 versus LPS group.

Figure 2 shows compound A’s anti-inflammatory activity. The expression of iNOS and inflammatory cytokines release were detected in LPS-stimulated RAW264.7 cells. As shown in Figure 3, compound A could decrease the expression of iNOS and the production of cytokines, such as NO, IL-1β, and IL-6, but increased COX2 expression. Meanwhile, the immunofluorescence results of iNOS and COX-2 reached the same conclusion in terms of protein expression. The results indicte that compound A could inhibit macrophage polarization to M1-type.

### 2.4. Compound A Inhibited Protein Expression in IL-4 Stimulated M2-Type Macrophages

**Figure 4:**
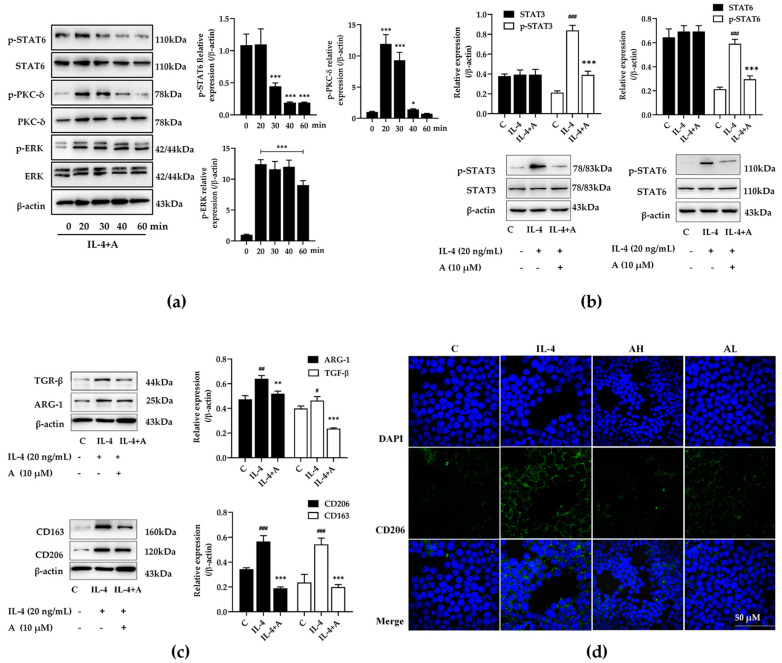
Compound A inhibited macrophages for polarized M2 phenotype in IL-4-stimulated RAW264.7 cells. (**a**) After treating with IL-4 (20 ng/mL) for 24 h, the expression and phosphorylation of PKC δ, ERK, and STAT6 were detected 0–60 min after compound A (10 μmol·L^−1^) treatment. Statistical significance was represented as follows: * *p* < 0.05, *** *p* < 0.001 versus 0 min group. (**b**) The expression and phosphorylation of STAT3 and STAT6 were detected 24 h after compound A treatment. Statistical significance was represented as follows: ^###^ *p* < 0.001 versus C group; *** *p* < 0.001 versus IL-4 group. (**c**) The expression of TGF-β, CD163, CD206, ARG-1 was detected. (**d**) Representative images of immunofluorescence staining of CD206 (green) and DAPI (blue) of IL-4-induced RAW264.7 cells. Statistical significance was represented as follows: ^#^ *p* < 0.05, ^##^ *p* < 0.01, ^###^ *p* < 0.001 versus C group; ** *p* < 0.01, *** *p* < 0.001 versus IL-4 group.

As reported, the activation of PKC could inhibit the polarization of tumor-associated M2 macrophages [15]. It has been confirmed in previous experiments that compound A could inhibit the polarization of M1-type macrophages. We further investigated how compound A regulates the polarization state of macrophages when cells exhibit the M2 phenotype. Firstly, RAW264.7 cells were treated with IL-4 and stimulated for 36 h to establish a M2 macrophage model. The expression and phosphorylation of PKC δ, STAT6, and ERK were detected after compound A treatment (Figure 4a). Compared to the control group, compound A could promote PKC δ and ERK activation, and inhibited STAT6 phosphorylation. To analyze the effect of compound A on these proteins for 24 h, as shown in Figure 4b, the activation of STAT3 and STAT6 were upregulated in IL-4-stimulated macrophages, but were inhibited in the compound A co-treatment group. Meanwhile, compound A downregulated the expression of ARG-1, CD163, TGF-β, and CD206 in IL-4-stimulated RAW264.7 cells (Figure 4c). Similarly, immunofluorescence staining of CD206 showed the same tendency (Figure 4d). In sum, compound A could inhibit the polarization of M2 macrophages.

### 2.5. Compound A Inhibited M2 Polarization of Macrophages under HepG2 and RAW264.7 Cells Co-Cultivation

**Figure 5:**
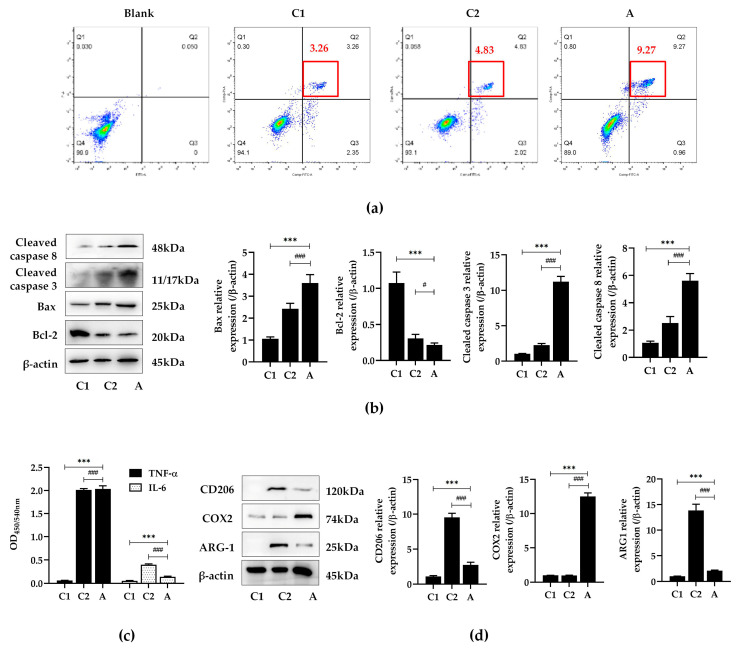
The effects of compound A treatment in HepG2 and RAW264.7 cell co-cultivations. (**a**) The flow cytometry image of cell apoptosis was analyzed by Flow cytometry. The red box represents double staining with Annexin V and PI, and the red text represents the proportion of double stained cells. C1: HepG2 cells; C1: HepG2 and RAW264.5 cell co-cultivations; A: HepG2 and RAW264.5 co-cultivations were treated with 10 μmol∙L^−1^ of compound A. Cells were treated for 48 h. (**b**) The expression of Bcl-2, Bax, cleaved caspase 3, and 8 were detected by Western blotting. Data are expressed as mean ± standard deviations. *** *p* < 0.001 versus C1 group; ^#^ *p* < 0.05, ^###^ *p* < 0.001 versus C2 group. (**c**) The levels of TNF-α and IL-6 were detected by ELISA kits. Data are expressed as mean ± standard deviations. *** *p* < 0.001 versus C1 group; ^###^ *p* < 0.001 versus C2 group. (**d**) The expression of ARG-1, COX2, and CD206 were detected by Western blotting. Data are expressed as mean ± standard deviations. *** *p* < 0.001 versus C1 group; ^###^ *p* < 0.001 versus C2 group.

In order to further investigate the effect of compound A on regulation the polarization of macrophages and anti-HepG2 cells, co-cultivations of the two cells were continuously treated for 48 h with compound A. Compared with the HepG2 cell group, the apoptosis rates of the co-cultivation group and the compound A group were upregulated; rates in the compound A group were the most significant (Figure 5a). In addition, compound A could significantly increase the level of Bax, and cleaved caspase 3 and 8 (Figure 5b). The results indicate that compound A promoted the apoptosis of HepG2 cells in co-cultivation. Compared with the HepG2 cell group and the co-cultivation group, compound A decreased the level of IL-6 and decreased the expression of ARG-1 and CD206, but increased COX2 (Figure 5c,d). These results indicate that compound A could promote the apoptosis of HepG2 cells and inhibit macrophage polarization.

## 3. Discussion

Macrophages are a highly heterogeneous cell population that exhibit unique phenotypes and functions in the complex microenvironment in vivo [20]. Macrophage polarization plays a key role in regulating the body’s immune response and metabolism; if it is disordered, it may cause disease. When inflammation occurs, macrophage polarization can initiate at any point in the inflammatory process. Several signaling pathways are involved in M1 and M2 macrophage polarization, such as TLR/NF-κB, JAK/STAT, and so on [21,22]. Multiple signaling molecules are initiated upon LPS binding to TLRs in macrophages, leading predominantly to the activation of protein kinase Cs (PKCs) and ERK 1/2 [23] or NF-κB [24]. In M2-polarization macrophages, it was demonstrated by Arruda S that IL-4 binding to IL-4R on human monocytes could cause PKC activation and translocation to a nuclear fraction [25]. In turn, feedback from these downstream signaling proteins activated a variety of transcription factors that coordinated the induction of genes encoding inflammatory mediators to promote macrophage polarization. Then the homeostasis of macrophages was disrupted, promoting disease progression.

**Figure 6:**
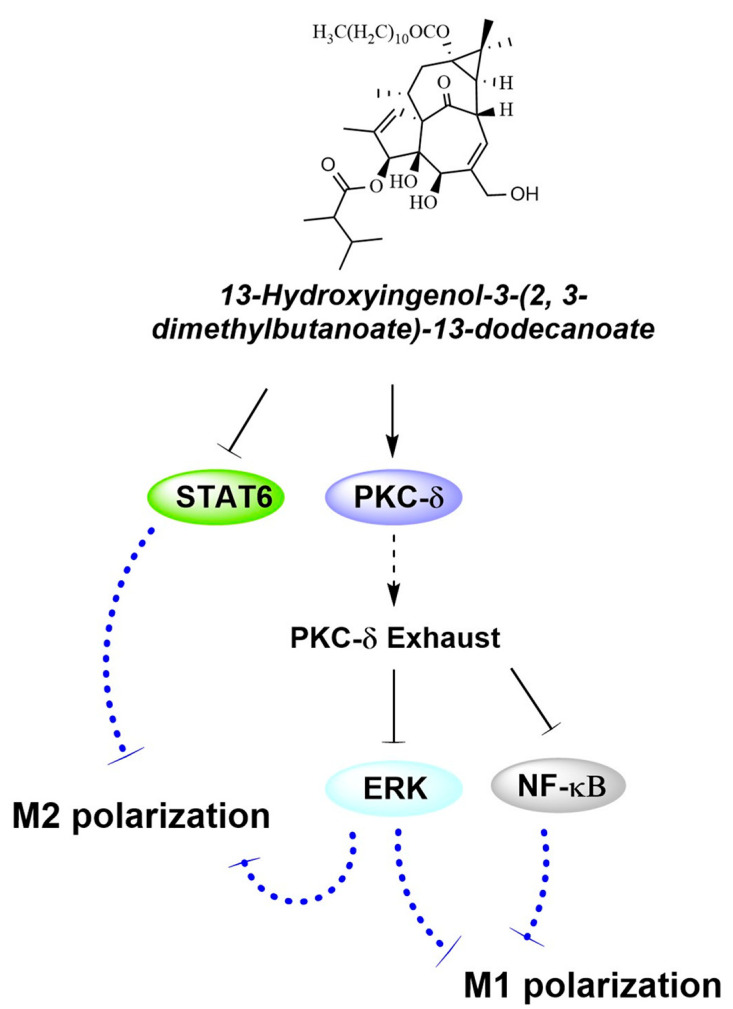
The possible mechanism of compound A on the polarization of macrophage.

Balancing the homeostasis of macrophages plays a key role in the treatment of inflammation diseases [26,27]. M1 macrophages are primarily anti-tumor and immunity-activating, but continuous inflammation promotes tissue damage. The regulation of M1/M2 macrophages polarization may be valuable in the treatment of diseases. Contrary to the classic anti-tumor effect of PKC activators, in the present research, we found that compound A promoted the apoptosis of HepG2 cells, conversely regulating PKC δ-ERK and STAT signaling pathways to inhibit M1 and M2 macrophage polarization. These might be the possible mechanisms of compound A mediating macrophage polarization by signaling multiple pathways, exerting the anti-tumor immunity effect (Figure 6). However, the present research only discussed the role of compound A on macrophage polarization regulation and its anti-tumor and immunomodulatory mechanisms under co-acculturation. Further research is needed on the role and mechanism of compound A in vivo.

## 4. Materials and Methods

### 4.1. Plant Extraction and Compound Isolation

*Euphorbia kansui* was collected in Linfen, Shanxi in May 2019, and was identified by Professor Guo Shiming, Yunnan Academy of Traditional Chinese Medicine. The specimen is preserved in the Resource Medicinal Chemistry Laboratory of the School of Life Science and Technology, Kunming University of Science and Technology, and the specimen number is: GS201905.

Compound A was extracted from the air-dried and powdered roots of *E. kansui*. The detailed separation process and spectroscopic data of compound A were described previously [18].

### 4.2. Reagents

Compound A was extracted and purified from *E. kansui* and supplied at 95% purity by Key Laboratory of resources and medicinal chemistry, Kunming University of Science and Technology. The structure of the compound was identified by Jianchun Li (Kunming University of Science and Technology). The voucher specimen of the compound (KMUST 20190301) was deposited at the Laboratory of Phytochemistry, Kunming University of Science and Technology. Compound A was subsequently made up to a stock of 50 mmol/L in dimethyl sulfoxide (DMSO, Aladdin, Riverside, CA, USA, J1914010), then the drug was stored at 4 °C. Bcl-2, Bax, ERK, and phospho-ERK were bought from CST (Cell Signaling Technology, Danvers, MA, USA), β-actin (Servicebio, Wuhan, China), ARG-1, CD163 (Beyotime, Shanghai, China), CD206 (Invitrogen-Thermo Fisher, Carlsbad, CA, USA). Cleaved caspase 8 was purchased from Affinity (Nanjing, China); the FITC rabbit antibody from ZhongshanJinqiao Biotechnology (Beijing, China); DAPI from Beyotime (Beijing, China); and the Annexin V/PI kits from BD (Franklin Lakes, NJ, USA).

### 4.3. Cell Culture and Model Established

Mouse macrophage cell line RAW264.7 was incubated in a DMEM medium (Gibco, Carlsabad, CA, USA) supplemented with 10% fetal bovine serum (FBS, Gibco), 10,000 U/mL penicillin, and 10,000 μg/mL streptomycin. RAW264.7 cells were treated with 1 μg/mL Lipopolysaccharide (LPS, Sigma, Saint Louis, MO, USA, 12181202) to promote the polarization of macrophages towards M1 or 20 ng/mL interleukin 4 (IL-4, Beyotime, Shanghai, China) to promote the polarization of macrophages towards M2.

### 4.4. MTT Assay

8 × 10^4^ cells/well were incubated in 96T wells for 24 h, and the cells were treated with a drug-containing medium for a specified time. Cells were added 20 μL 0.5% MTT (Sigma, USA) to each well, continued to incubate at 37 °C for 3.5 h, then the medium was discarded and 150 μL of DMSO was added to each well to dissolve the purple formazan. The cells were shaken for 15 min at room temperature and read at 490 nm. Cell viability (%) = (OD_control_ − OD_sample_)/(OD_control_ − OD_blank_) × 100%. IC_50_ (half maximal inhibitory concentration) was calculated by SPSS 27.0 software.

### 4.5. Flow Cytometry

Cells with the relevant surface antibodies were incubated at 4 °C for 30 min without light. The stained cells were incubated with Annexin V/PI kits from BD. The experiment was conducted according to the instructions. Data were analyzed on FlowJo V10 software.

### 4.6. Fluorescence Staining

RAW264.7 cells were fixed with 500 μL/well 4% paraformaldehyde for 20 min at room temperature, washed with 1 mL/well PBS five times, then incubated with anti-iNOS, anti-COX-2, or anti-CD206 antibodies at 4 °C overnight, separately. The cells were washed with 1 mL/well PBS five times, then treated with the secondary antibody (FITC-Goat anti-rabbit IgG antibody, 1:200-fold dilution) and DPAI (100 μL/well). The cells were observed with a laser confocal microscope. The stained images were captured under a laser scanning confocal microscope (Nikon A1 AIR MP+, Tokyo, Japan).

### 4.7. Measurement of Nitric Oxide and Cytokines

The isolates were evaluated for their inhibitory effect on NO production in LPS-induced RAW264.7 cells. The iNOS inhibitor (NG-monnomycin-L-arginine, L-NMMA) was selected as the positive control. NO was evaluated by absorbance-based detection on nitrite accumulated in the culture medium. The levels of cytokines (IL-1β, IFN-γ, IL-10, TNF-α, and IL-6) in the cell supernatant were determined using an ELISA kit (Novus Biologicals, Centennial, CO, USA) according to the manufacturer’s instructions. The absorbance was read at OD_450nm_/OD_540nm_ in SpectraMax340PC microplate spectrophotometer (Molecular Devices, San Jose, CA, USA).

### 4.8. Western Blotting Analysis

Protein samples were prepared using a RIPA lysis buffer. The concentration of the protein was analyzed using nucleic acid and protein microanalyzer (Molecular Devices, San Jose, CA, USA). SDS-PAGE gel electrophoresis was performed to separate the proteins, which then were electro-transferred onto a PVDF membrane. The membranes were blocked with 5% non-fat milk for 2 h and incubated with primary antibodies and secondary antibodies. The protein bands were detected by the ECL and were exposed to the TANON gel imager (Shanghai, China). The results were analyzed by Image J 180 software.

### 4.9. Protein Kinase Inhibitor

In order to investigate the effects of PKC activation on the effects of drug treatment, 150 nmoL/L bisindolylmaleimide (BIS) was used. BIS (Beyotime, Shanghai, China) is a potent inhibitor of PKC α/β/γ and PKC δ/ε. This agent was stored at −80 °C in DMSO and cells were pre-incubated with the inhibitor for 4 h prior to being treated with compound A.

### 4.10. Statistical Analysis

Data are presented as means with standard deviations (SD). Independent two-sample *t*-tests compared differences between two groups, and one-way ANOVA with the least significant difference (LSD) test for post-host comparisons compared differences between three or four groups. A *p*-value below 0.05 indicated statistical significance. Statistics were analyzed with SPSS 21.0 software (SPSS Inc., Chicago, IL, USA).

## 5. Conclusions

In conclusion, compound A could not only promote the apoptosis of HepG2 cells but also inhibit the M1 and M2 phenotype macrophages by PKC δ/ERK and STAT signaling pathways. Under co-cultivation, compound A could further promote the apoptosis of HepG2 cells and inhibit macrophage polarization to M2 phenotype in vitro. These results showed that compound A might play an important role in balancing phenotype macrophages, which this might be its immunomodulatory mechanism (Figure 6).

## Figures and Tables

**Table 1:** Cell viability of A549, MCF-7, and HepG2 cells treated with compound A ($\bar{x}$ ± s, n = 3).

Drug	A549 IC_50_ (μM)	MCF-7 IC_50_ (μM)	HepG2 IC_50_ (μM)
Control	-	-	-
Adr ^1^	5.98 ± 2.06	3.31 ± 1.01	4.37 ± 1.21
Compound A	21.97 ± 5.01	27.12 ± 3.34	20.97 ± 4.53

^1^ Adr: adriamycin.

**Table 2:** Cell viability of RAW264.7 cells treated with compound A and the NO inhibition activities on LPS-stimulated RAW264.7 ($\bar{x}$ ± s, n = 3).

Compounds	RAW264.7	
	CC_50_^1^ (μM)	IC_50_ ^2^ (μM)
Control	-	-
LPS ^3^	-	-
Compound A	20.34 ± 3.62	0.57 ± 0.07
l-NMMA^4^	>50	21.80 ± 3.21

^1^ Cell survival rate (%) = (OD_drug_ − OD_blank_)/(OD_control_ − OD_blank_) × 100%. CC_50_ value refers to the half inhibitory of RAW264.7 cells. ^2^ NO inhibition rate (%) = (OD_LPS_ − OD_drug_)/(OD_LPS_ − OD_control_) × 100%. The IC_50_ value refers to the inhibitory nitric oxide production of LPS-stimulated RAW264.7 cells. ^3^ LPS: Lipopolysaccharide. ^4^ l-NMMA: NG-monnomycin-l-arginine, iNOS inhibitor. Positive control in RAW264.7.

## Data Availability

The datasets used in the present study are available from the corresponding authors upon reasonable request.

## References

[1] Feng X.Y., Chen B.C., Li J.C., Li J.M., Li H.M., Chen X.Q., Liu D., Li R.T. (2021). Gansui-Banxia Decoction extraction inhibits MDSCs accumulation via AKT/STAT3/ERK signaling pathways to regulate antitumor immunity in C57bl/6 mice. Phytomedicine.

[2] Huo M., Wang T., Li M., Li N., Chen S., Xiu L., Yu X., Liu H., Zhong G. (2024). Gansui Banxia decoction modulates immune-inflammatory homeostasis to ameliorate malignant ascites in rats. Phytomedicine.

[3] Meng X.H., Wang K., Chai T., Guo Z.Y., Zhao M., Yang J.L. (2020). Ingenane and jatrophane diterpenoids from *Euphorbia kansui* and their antiproliferative effects. Phytochemistry.

[4] Wang S., Li J., Liu D., Yang T., Chen X., Li R. (2021). Ingenane and jatrophane-type diterpenoids from *Euphorbia kansui* with multidrug resistance reversal activity. Phytochemistry.

[5] Chen C.S., Pan B.Y., Tsai P.H., Chen F.Y., Yang W.C., Shen M.Y., Kansuinine A. (2021). Ameliorates Atherosclerosis and Human Aortic Endothelial Cell Apoptosis by Inhibiting Reactive Oxygen Species Production and Suppressing IKKbeta/IkappaBalpha/NF-kappaB Signaling. Int. J. Mol. Sci..

[6] Silva V.A.O., Rosa M.N., Miranda-Goncalves V., Costa A.M., Tansini A., Evangelista A.F., Martinho O., Carloni A.C., Jones C., Lima J.P. (2019). Euphol, a tetracyclic triterpene, from Euphorbia tirucalli induces autophagy and sensitizes temozolomide cytotoxicity on glioblastoma cells. Investig. New Drugs.

[7] Wang P., Lu P., Qu X., Shen Y., Zeng H., Zhu X., Zhu Y., Li X., Wu H., Xu J. (2017). Reactivation of HIV-1 from Latency by an Ingenol Derivative from *Euphorbia Kansui*. Sci. Rep..

[8] Oh S., Oh H.W., Lee H.R., Yoon S.Y., Oh S.R., Ko Y.E., Yoo N., Jeong J., Kim J.W. (2016). Ingenane-type diterpene compounds from *Euphorbia kansui* modulate IFN-gamma production through NF-kappaB activation. J. Sci. Food Agric..

[9] Zhang Y., Lou J.W., Kang A., Zhang Q., Zhou S.K., Bao B.H., Cao Y.D., Yao W.F., Tang Y.P., Zhang L. (2020). ameliorate malignant ascites by modulating gut microbiota and related metabolic functions. J. Ethnopharmacol..

[10] Zhang Q., Li Z.L., Zhang Y., Wang K., Zhang M., Chen P.D., Yao W.F., Tang Y.P., Wu J.H., Zhang L. (2020). Effect of the vinegar-process on chemical compositions and biological activities of *Euphorbia kansui*: A review. J. Ethnopharmacol..

[11] Hampson P., Chahal H., Khanim F., Hayden R., Mulder A., Assi L.K., Bunce C.M., Lord J.M. (2005). PEP005, a selective small-molecule activator of protein kinase C, has potent antileukemic activity mediated via the delta isoform of PKC. Blood.

[12] Ersvaer E., Kittang A.O., Hampson P., Sand K., Gjertsen B.T., Lord J.M., Bruserud O. (2010). The protein kinase C agonist PEP005 (ingenol 3-angelate) in the treatment of human cancer: A balance between efficacy and toxicity. Toxins.

[13] Kawano T., Inokuchi J., Eto M., Murata M., Kang J.H. (2022). Protein Kinase C (PKC) Isozymes as Diagnostic and Prognostic Biomarkers and Therapeutic Targets for Cancer. Cancers.

[14] Donohoe F., Wilkinson M., Baxter E., Brennan D.J. (2020). Mitogen-Activated Protein Kinase (MAPK) and Obesity-Related Cancer. Int. J. Mol. Sci..

[15] Cheng Y., Zhu Y., Xu J., Yang M., Chen P., Xu W., Zhao J., Geng L., Gong S. (2018). PKN2 in colon cancer cells inhibits M2 phenotype polarization of tumor-associated macrophages via regulating DUSP6-Erk1/2 pathway. Mol. Cancer.

[16] Wang W., Qian B., Zhao C., Peng M., Liu L., Xie M., Peng N., He Q., Ying S., Zhu Y. (2022). Sublytic C5b-9 Induces CCL3/4 Production and Macrophage Accumulation in Thy-1N Rats via PKC-alpha/p65/IRF-8 Axis. Int. J. Biol. Sci..

[17] Simpson R.U., O’Connell T.D., Pan Q., Newhouse J., Somerman M.J. (1998). Antisense oligonucleotides targeted against protein kinase Cbeta and CbetaII block 1,25-(OH)2D3-induced differentiation. J. Biol. Chem..

[18] Li J.-C., Li S.-Y., Tang J.-X., Liu D., Feng X.-Y., Rao K.-R., Zhao X.-D., Li H.-M., Li R.-T. (2022). Triterpenoids, steroids and other constituents from *Euphorbia kansui* and their anti-inflammatory and anti-tumor properties. Phytochemistry.

[19] Liu Q., Li W., Huang L., Asada Y., Morris-Natschke S.L., Chen C.H., Lee K.H., Koike K. (2018). Identification, structural modification, and dichotomous effects on human immunodeficiency virus type 1 (HIV-1) replication of ingenane esters from *Euphorbia kansui*. Eur. J. Med. Chem..

[20] Wang C., Ma C., Gong L., Guo Y., Fu K., Zhang Y., Zhou H., Li Y. (2021). Macrophage Polarization and Its Role in Liver Disease. Front. Immunol..

[21] Wang H., Yung M.M.H., Ngan H.Y.S., Chan K.K.L., Chan D.W. (2021). The Impact of the Tumor Microenvironment on Macrophage Polarization in Cancer Metastatic Progression. Int. J. Mol. Sci..

[22] Saeedifar A.M., Mosayebi G., Ghazavi A., Bushehri R.H., Ganji A. (2021). Macrophage polarization by phytotherapy in the tumor microenvironment. Phytother. Res..

[23] Lu Z., Liu D., Hornia A., Devonish W., Pagano M., Foster D.A. (1998). Activation of protein kinase C triggers its ubiquitination and degradation. Mol. Cell Biol..

[24] Zhang L., Zhao S., Wang Y. (2024). Diannexin alleviates myocardial ischemia-reperfusion injury by orchestrating cardiomyocyte oxidative damage, macrophage polarization and fibrotic process by TLR4-NF-kB-mediated inactivation of NLRP3 inflammasome. Int. Immunopharmacol..

[25] Song Y.N., Lee J.W., Ryu H.W., Lee J.K., Oh E.S., Kim D.Y., Ro H., Yoon D., Park J.Y., Hong S.T. (2023). Black Ginseng Extract Exerts Potentially Anti-Asthmatic Activity by Inhibiting the Protein Kinase Ctheta-Mediated IL-4/STAT6 Signaling Pathway. Int. J. Mol. Sci..

[26] Kloosterman D.J., Akkari L. (2023). Macrophages at the interface of the co-evolving cancer ecosystem. Cell.

[27] Li Z., Li D., Chen R., Gao S., Xu Z., Li N. (2023). Cell death regulation: A new way for natural products to treat osteoporosis. Pharmacol. Res..

